# SIP30 involvement in vesicle exocytosis from PC12 cells

**DOI:** 10.1016/j.bbrep.2023.101614

**Published:** 2023-12-15

**Authors:** Ning Guo, Lei Yu

**Affiliations:** Department of Genetics, and Center of Alcohol & Substance Use Studies, Rutgers University, Piscataway, NJ, 08854, USA

**Keywords:** SIP30, Exocytosis, Synaptic vesicles, PC12 cells, Neuropathic pain

## Abstract

SNAP25 (synaptosome-associated protein of 25 kDa) is a core SNARE (soluble N-ethylmaleimide-sensitive factor attachment receptor) protein; and the interaction between SNAP25 and other SNARE proteins is essential for synaptic vesicle exocytosis. Identified as a SNAP25 interacting protein, SIP30 (SNAP25 interacting protein at 30 kDa) has been shown to modulate neuropathic pain behavior, and is potentially involved in the cellular process of vesicle exocytosis. Previous study demonstrated that using a vesicle secretion assay in PC12 cells, anti-SIP30 siRNA reduced vesicle exocytosis. We investigated vesicle exocytosis from PC12 cells with FM1-43 fluorescence dye, and demonstrated that anti-SIP30 siRNA reduced the pool of releasable vesicles and the rate of vesicle exocytosis, without affecting the endocytosis and recycling of the exocytosed vesicles. The results show that SIP30 is involved in vesicle exocytosis, suggesting a potential mechanism of SIP30 modulation of neuropathic pain.

## Highlights

• SIP30 is a SNARE protein involved in neuropathic pain modulation.
• Inhibition of SIP30 in PC12 cells reduced the pool of releasable vesicles and the rate of vesicle exocytosis, without affecting the endocytosis and recycling of the exocytosed vesicles.
• This suggests SIP30 involvement in vesicle exocytosis, thereby modulating neuropathic pain.

## 1 Introduction

The evoked release of neurotransmitters from neurons and neurosecretory cells is important for communication between neurons and/or other types of cells, and is a highly orchestrated cellular process that involves the participation and interactions of many proteins [[1], [2], [3], [4]]. In the nervous system, neurotransmitters are accumulated and stored in synaptic vesicles, and are released through the fusion of synaptic vesicle membrane and presynaptic plasma membrane upon the arrival of the releasing signals [[5], [6], [7]]. SNARE (soluble N-ethylmaleimide-sensitive factor attachment receptor) proteins are of fundamental importance in forming the machinery for fusion between vesicle membrane and cellular membrane [[8], [9], [10]]. SNARE proteins can be categorized as v-SNAREs (vesicle membrane associated SNARE proteins, which include synaptobrevin family proteins) and t-SNAREs (target membrane associated SNARE proteins, which include syntaxin family proteins and SNAP25 family proteins). SNARE proteins possess the defining SNARE motifs, which are essentially coiled-coil domains with about 70 amino acid residues that usually mediate protein-protein interactions [11]. In neurons, the three SNARE proteins, synaptobrevin2, syntaxin1a and SNAP25, form a ternary complex, which is known as SNARE complex, via their SNARE motifs, and bring the opposing vesicle membrane and cell membrane into juxtaposition, therefore providing a scaffold for membrane fusion [4,12,13]. Proteins that interact with SNARE proteins are shown to participate in the cellular process of evoked vesicle exocytosis, such as Munc18, complexin1, *etc.* [4,[14], [15], [16]].

SIP30 was identified to interact with SNA25 in a yeast two-hybrid screening for SNAP25 interacting proteins [17]. Biological function of SIP30 has been associated with neuropathic pain [[18], [19], [20]]. Analysis of the amino acid sequence of SIP30 indicated a putative coiled-coil domain that potentially interferes with the assembly of the SNARE complex [1,21]. SNARE proteins play a pivotal role in vesicle exocytosis [9,22]. Several other proteins that interact with SNAP25 and possess coiled-coil domains were discovered to regulate synaptic vesicle exocytosis [[23], [24], [25]], which supports the hypothesis that, as a SNAP25 interacting protein, SIP30 may play a similar regulatory role in synaptic vesicle exocytosis.

PC12 cells that originate from rat adrenal chromaffin cells have been studied as a model system of both neurosecretion from adrenal chromaffin cells and neurotransmitter from neurons [26]. In the current study, the evoked exocytosis from PC12 cells was used as a model system to investigate the involvement of SIP30 in vesicle exocytosis. PC12 cells contain the complete machinery for vesicle exocytosis, therefore enabling genetic manipulations to study the functions of individual player by either over-expression or expression inhibition. As shown in our previous study, inhibition of SIP30 by siRNA in PC12 cells resulted in much reduced neurotransmitter release, suggesting a role of SIP30 in vesicle exocytosis [18]. Vesicle exocytosis is a dynamic process that involves multiple steps; therefore, quantitative fluorescence measurement of FM1-43 turnover was used to determine the specific steps in which SIP30 participates.

## 2 Materials and methods

### 2.1 PC12 cell culture

PC12 cells were obtained from ATCC (CRL-1721). PC12 cells were maintained in DMEM high glucose cell culture medium (D6171, SigmaAldrich, St. Louis, USA) supplemented with 6.5 % Fetal Bovine Serum, 6.5 % Horse Serum, and 2 mM l-glutamine at 37 °C with 5 % CO_2_. All cell culture plates and dishes were coated with 0.01 % Poly-d-Lysine (molecular weight 70K–150K, P0899, SigmaAldrich, St. Louis, USA). Cells were subcultured every 7 days with split ratio of 1:8.

### 2.2 Vesicle exo- and endo-cytosis kinetics using FM1-43

PC12 cells were seeded into each 60 mm cell culture dishes at 4 × 10^6^ cells per dish. Cells were transfected with either control siRNA or SIP30 siRNA as previously described [18] without hGH expressing plasmid. The sequence specificity of SIP30 oligonucleotide inhibition has been previously reported [18], with treatment-induced reduction of both SIP30 mRNA and protein. We further confirmed the specific reduction of SIP30 protein levels by Western blotting (data not shown). Forty-eight hours after transfection, PC12 cells were harvested and replated at 5 × 10^4^ cells/dish into poly-d-lysine coated 35 mm glass bottom petri dishes (P35GC-0-14-C, MatTek Corporation, USA). Twenty-four hours after replating, cells were subjected to one of the following experimental procedures specified as Experiment #1, Experiment #2 and Experiment #3. FM1-43 (T-35356, Invitrogen, USA) was first dissolved in DMSO at 10 mM as the stock. PSS buffers (low K^+^ (145 mM NaCl, 5.6 mM KCl, 2.2 mM CaCl_2_. 0.5 mM MgCl_2_, 5.6 mM Glucose, 15 mM HEPES, PH 7.4) or high K^+^ (95 mM NaCl, 56 mM KCl, 2.2 mM CaCl_2_. 0.5 mM MgCl_2_, 5.6 mM Glucose, 15 mM HEPES, PH 7.4)) containing 5 μM FM1-43 were freshly made shortly before use from the 10 mM stock.

PC12 cells in glass bottom dish were gently washed with PBS. Then the dish was mounted onto PTI RatioMaster system, and PC12 cells were locally superfused with buffers as determined by experimental procedure with a custom-made 4-channel superfusion system with a flow rate of about 1 ml/min. A xenon arc lamp provided high intensity, continuous broadband illumination, and the wattage was set at 75 W. The excitation wave length for FM1-43 fluorescence was set to 475 nm. Fluorescence signal was detected with an analogue/digital photon counting detector through green filter, and fluorescence intensity was converted to arbitrary numbers. Data acquisition was with FeLix software that came with the system. All recorded fluorescence intensity numbers were normalized by the number of cells presented in the recording window.

Three experimental paradigms were designed based on published methods [27] with modifications.

Experiment#1: To examine the exocytosis of recycling vesicles, exocytosis from PC12 cells was stimulated by superfusion with high K^+^ PSS buffer containing 5 μM FM1-43 for various time periods (30 s, 60 s, 90 s, and 120 s). After stimulation, the cells were superfused for an additional 120 s with low K^+^ buffer containing 5 μM FM1-43. Therefore, during this loading session, FM1-43 was taken into the cells by endocytosis of exocytosed vesicles. Then the cells were thoroughly washed for 5 min with low K^+^ buffer without FM1-43. After wash, these FM1-43 loaded cells were stimulated to unload the trapped FM1-43 with 3 min superfusion of High K^+^ buffer, and the differences in fluorescence intensities (FΔt) before and after the unloading were taken as the amount of FM1-43 trapped in the recycling vesicles, which reflected the amount of recycling vesicles being labeled with FM1-43. Data are presented as FΔt vs. Δt, therefore, by varying the length of depolarization, the rate and the maximum level of exocytosis could be measured.

Experiment#2: To examine the kinetics of recycling vesicle endocytosis, PC12 cells were stimulated by superfusion with high K^+^ buffer for 60 s without FM1-43, and then low K^+^ buffer for various relaxation time periods (0 s, 30 s, 60 s, 90 s, and 120 s). FM1-43 was added into the low K^+^ superfusion buffer to 5 μM after the relaxation for 120 s to label the vesicles that were endocytosed after relaxation period. Then the cells were thoroughly washed for 5 min with low K^+^ buffer without FM1-43. After wash, these FM1-43 loaded cells were stimulated to unload the trapped FM1-43 with 3 min superfusion of High K^+^ buffer. The differences in fluorescence intensities (FΔt) before and after the final unloading were taken as representative of the amount of vesicles being endocytosed after the relaxation period. Data are presented as FΔt/F0 vs Δt, therefore, by varying the length of relaxation, the rate of endocytosis could be measured.

Experiment#3: To examine the replenishment of releasable vesicles from the endocytosed recycling vesicles, PC12 cells were stimulated to exocytose with high K^+^ buffer in the presence of 5 μM FM1-43 for 60 s. Therefore, the vesicles being endocytosed during this 60 s were labeled with FM1-43. After loading, cells were continuously superfused with high K^+^ buffer for various chasing period without FM1-43 (0 s, 30 s, 60 s, 90 s, and 120 s), so that labeled vesicles became releasable for the second round of exocytosis during the chasing period were de-stained. After the chasing period, cells were thoroughly washed for 5 min with low K^+^ buffer, and remaining labeled vesicles were then unloaded by 3 min superfusion with high K^+^ buffer. The differences in fluorescence intensities (FΔt) before and after the final unloading were taken as the amount of labeled vesicles retained after chasing. Data are presented as (F0-FΔt)/F0 vs Δt, so that a decrease in the fluorescent intensity along time, which was shown as a rise on the curve, represented the re-entry of endocytosed vesicle into readily releasable pool.

### 2.3 Statistic analysis

Data of experiments with FM1-43 were analyzed with two-way ANOVA with time and treatment as parameters.

## 3 Results

### 3.1 The pool of releasable vesicles in PC12 cells and the releasing rate constant of releasable vesicles were diminished by SIP30 siRNA transfection

To assess the size of releasable vesicle pool and the release kinetics of releasable vesicles, PC12 cells were incubated with high K^+^ buffer for different durations, and the vesicles were labeled as described in materials and methods. The longer the duration of depolarization, the more vesicles were exocytosed and subsequently endocytosed, thus the more FM1-43 was internalized and the higher the fluorescence intensity was trapped within the cells. The fluorescence intensity of recycling vesicles was plotted against the duration of the depolarization stimulation to determine the size of the recycling vesicle pool and also to estimate the releasing rate of the recycling vesicles.

**Fig. 1:**
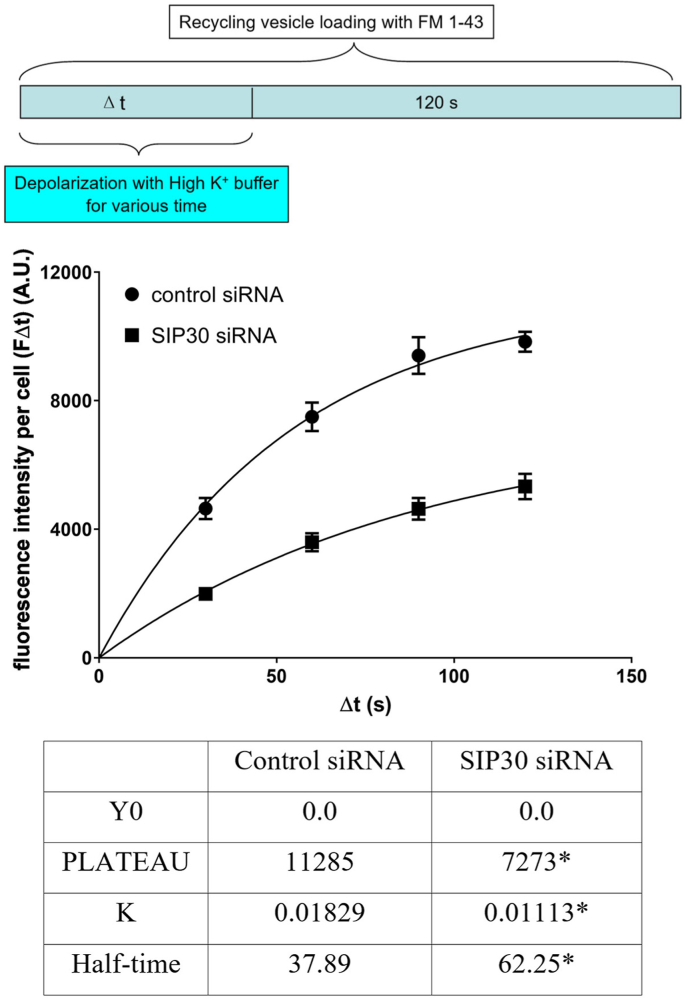
SIP30 siRNA transfection resulted in a reduction of the functional recycling vesicle pool, and reduced the rate of vesicle releasing. Cultured PC12 cells were transfected with either control siRNA or SIP30 siRNA. Seventy-two hr after transfection, experiment with FM1-43 was carried out at room temperature. Top panel: A schematic diagram illustrating the experimental design, highlighting (in cyan color) the step of varying depolarization time. Middle panel: A graph displaying the fluorescence intensity of recycling vesicles plotted against the duration of the depolarization stimulation, showing that the releasable vesicle pool was reduced to about 65 % in SIP30 siRNA treated PC12 cells (squares) compared with control group (circles). “Fluorescence intensity per cell” represented the amount of FM1-43 internalized by endocytosis of the vesicles, and was plotted as a function of depolarization duration (Δt). Data were fitted with one-phase exponential association curves. Bottom panel: A table showing the important parameters of the fitted exponential association curves. Data are presented as mean ± SEM, n = 6. In each recording, total fluorescence of 6–12 cells was measured and normalized by the number of cells. *, significantly different compared with the control as determined by ANOVA.

Data were fitted with one-phase exponential association curves (Fig. 1). The results showed that the size of the releasable vesicle pool was reduced to about 65 % in SIP30 siRNA treated PC12 cells compared with the control group, which is consistent with the human growth hormone secretion assay. SIP30 siRNA transfection also caused a nearly 40 % reduction in the releasing rate constant, as reflected by both the reduction in the rate constant K and increased time t_1/2_ for half of the releasable vesicles to be released (significant difference, two-way ANOVA).

### 3.2 The endocytosis of vesicles was not affected by SIP30 siRNA transfection

To investigate whether SIP30 participates in the endocytosis of vesicles subsequently after exocytosis, PC12 cells were depolarized with high K^+^ buffer for a fixed period of time, and then the recycling vesicles were labeled with FM1-43 after various duration of relaxation period, as described in materials and methods. The membrane that underwent endocytosis during the relaxation period was not labeled, whereas the membrane endocytosed during the labeling period was labeled. Therefore, the longer the relaxation period, the more vesicles were endocytosed without the presence of FM1-43, and thus the fewer the endocytosed vesicles were labeled with FM1-43. The fluorescence intensity of FM1-43 trapped in the endocytic vesicles was plotted against the relaxation duration in order to determine how fast the vesicles were endocytosed.

**Fig. 2:**
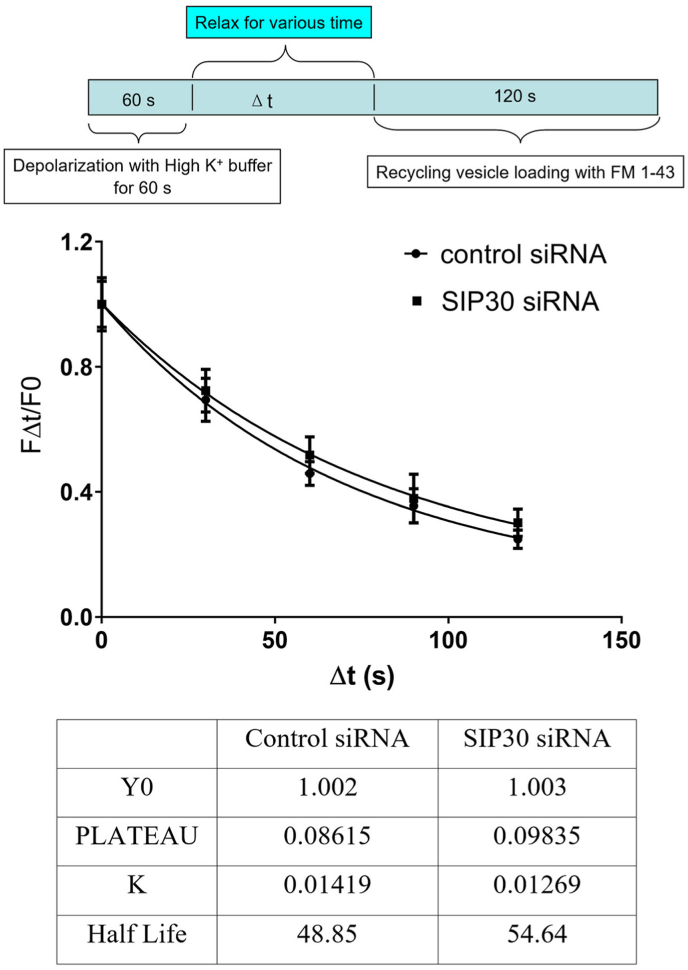
SIP30 siRNA transfection did not affect the endocytotic kinetics of recycling vesicles. Cultured PC12 cells were transfected with either control siRNA or SIP30 siRNA. Seventy-two hr after transfection, experiment with FM1-43 was carried out at room temperature. PC12 cells were depolarized with high K^+^ for 60s and waited for various duration (Δt) before FM1-43 was added to label the remaining endocytotic vesicles. Fluorescence intensity FΔt was normalized with F0 (Δt = 0) to account for the difference in maximum possible loading. Top panel: A schematic diagram illustrating the experimental design, highlighting (in cyan color) the step of varying relaxation time. Middle panel: A graph displaying the fluorescence intensity of FM1-43 trapped in the endocytic vesicles (normalized with the maximum loading of the group) plotted against the relaxation duration. Data were fitted with one-phase exponential decay curves. Bottom panel: A table showing the important parameters of the fitted exponential association curves. Data are presented as mean ± SEM, n = 6. In each recording, total fluorescence of 6–12 cells was measured and normalized by the number of cells.

To account for the differences in vesicle pool size between SIP30 siRNA and control siRNA treated groups, fluorescence intensities were normalized with the maximum loading of each respective group. Data from each group were fitted with one-phase exponential decay curve (Fig. 2), and the two curves of different treatment were not different from each other (two-way ANOVA analysis). In both groups, the t_1/2_ for endocytosis of recycling vesicle was about the same at roughly 50 s, and the over all shape of the curve overlapped. The result showed no differences in the rate constant between the two groups, which indicated that inhibition of SIP30 expression did not impact the endocytosis of recycling vesicles, thus indicating that SIP30 did not participate in endocytic machinery.

### 3.3 The reformation of fusion competent vesicles from the endocytosed vesicles was not affected by SIP30 siRNA transfection

The endocytosed membrane, in the form of vesicles, is recycled to replenish the pool of vesicles. To determine the time course of re-entry of the endocytosed vesicles into readily releasable pool, PC12 cells were stimulated with high K^+^ buffer with the presence of FM1-43 and the recycling vesicles were pulse labeled. The high K^+^ stimulation continued after the removal of FM1-43 for various chasing periods; therefore, the labeled vesicles that re-entered the readily releasable pool during the chasing period were released again and lost their fluorescence. The longer the chasing period, the more vesicles re-entered the readily releasable pool at the presence of high K^+^, and the less the retaining fluorescence, which represented the vesicles that were not ready for release after endocytosis.

**Fig. 3:**
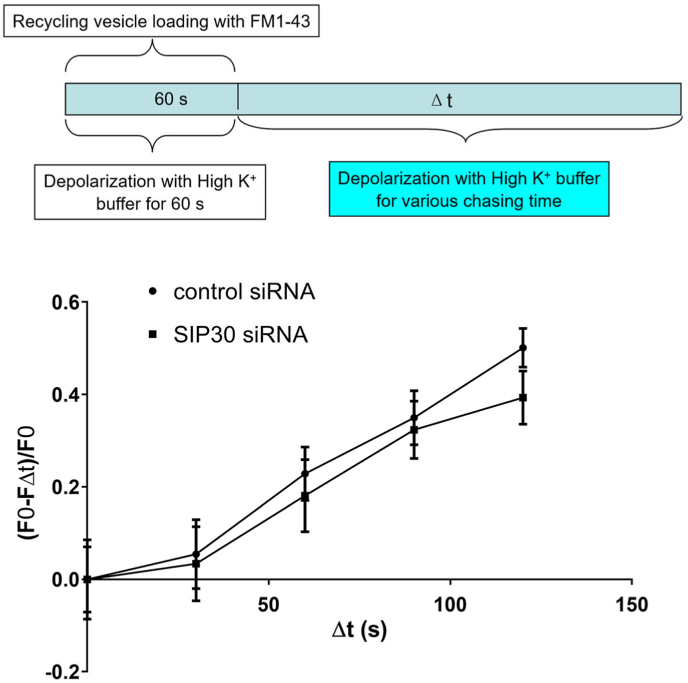
The kinetics of fusion competent vesicle reformation from endocytosed recycling vesicles was not affected by SIP30 siRNA transfection. Cultured PC12 cells were transfected with either control siRNA or SIP30 siRNA. Seventy-two hr after transfection, experiment with FM1-43 was carried out at room temperature. PC12 cells were depolarized with high K^+^ with the presence of FM1-43 for 60 s. Then FM1-43 was removed from incubation buffer, while the depolarization continued for various chasing period (Δt). Top panel: A schematic diagram illustrating the experimental design, highlighting (in cyan color) the varying chasing time. Bottom panel: A graph displaying the (F0-FΔt)/F0 ratio (the relative amount of internalized FM1-43 being released) plotted against the chasing duration. The (F0-FΔt)/F0 ratio indicates the amount of endocytosed vesicles being released again. Data are presented as mean ± SEM, n = 6. In each recording, total fluorescence of 6–12 cells was measured and normalized by the number of cells.

Data were normalized with maximum possible loading to account for the differences in vesicle pool size (Fig. 3). The curves for SIP30 and control siRNA transfected groups largely overlapped with each other, which indicated that SIP30 expression inhibition did not affect the reformation of the fusion competent vesicles from the endocytosed vesicles. Taken the 60 s vesicle loading period into consideration, the result further indicated that, in PC12 cells, the minimum time required for the reentry of the endocytosed vesicles into readily releasable vesicles was about 90 s under the experimental condition in this study.

## 4 Discussion

Vesicle exocytosis is a dynamic process. After exocytosis, the newly exposed membrane is retrieved by endocytosis in the form of endocytic vesicles to maintain a relatively constant cell surface and to recycle the vesicles, which functions to replenish the pool of releasable vesicles. Besides releasable vesicle pool, another pool of vesicles also exists and is referred as reserved pool or resting pool. These reserved vesicles function to replenish the releasable pool at much slower rate when the releasable vesicles are depleted by strong stimulation. The existence of different pools of vesicles and their functional significance has been extensively discussed [[28], [29], [30]]. In order to get better understanding of how SIP30 is involved in the cellular process, vesicle exocytosis and recycling in PC12 cells were investigated with fluorescence dye FM1-43, which is reversibly incorporated into but does not diffuse across the membrane, thus selectively labeling only the external leaflet of the lipid bilayer and the vesicular surface of the endocytic vesicles in the scenario of exo/endo-cytosis.

In PC12 cells, both dense core vesicles that contain catecholamine neurotransmitters and small synaptic like vesicles that contain acetylcholine exist. Although the transfected human growth hormone is specifically localized in dense core vesicles in PC12 cells, the membrane depolarization induces exocytosis of both types of vesicles; therefore, the internalization of FM1-43 by membrane endocytosis does not discriminate the different origins of endocytosed membrane. Study that compared the exocytotic mechanisms between acetylcholine and catecholamine containing vesicles in PC12 cells showed that these two types of vesicles shared many common mechanisms for exocytosis, such as the dependence on Ca^++^, ATP, and GTP [31]. Using radioactivity labeled acetylcholine and catecholamine as readout for exocytosis from PC12 cells, Schubert and Klier showed that the exocytosis of the two neurotransmitters from the two different types of vesicles followed similar kinetics [32]. Therefore, although the exact contribution of the internalized FM1-43 fluorescence by the two types of vesicles was unknown in this current study, the reduced internalization of FM1-43 in SIP30 siRNA transfected PC12 cells indicated a reduction in the capacity of vesicle exocytosis. In addition, the rate constant K for vesicle exocytosis is independent of the plateau; therefore, the reduction in rate constant K in SIP30 siRNA transfected PC12 cells (Fig. 1) could not be explained by the reduced availability of releasable vesicles, which indicated a direct involvement of SIP30 in the molecular machinery of vesicle exocytosis.

The kinetics of membrane endocytosis after exocytosis of vesicles from PC12 cells was also investigated with FM1-43. Although the total releasable vesicles were reduced by SIP30 siRNA treatment, the relative speed of endocytosis of exocytosed vesicle membrane was not changed, as reflected by similar rate constant K (Fig. 2) between SIP30 siRNA and control siRNA treated PC12 cells. This implied that SIP30 did not affect the membrane endocytosis.

In this current study, the recycling of membrane that was labeled with FM1-43 during the loading session was observed in PC12 cells, as demonstrated by the chasing experiment (Fig. 3). The recycling of dense core vesicles has been reported in adrenal chromaffin cells. In bovine adrenal chromaffin cells, dense core vesicles that were loaded with fluid phase marker horse-radish peroxidase (HRP) by stimulated exocytosis were ready for the next round of releasing within 5 min after loading. In addition, the amount of HRP secreted did not change when waited longer (after 1 h), which indicated that all the endocytosed vesicles containing HRP were ready for the second round of release [33]. In mouse adrenal chromaffin cells, stimulation with action potentials at 0.5 Hz delivered by a patch-clamp electrode induced internalization of FM1-43, which was supplemented in the bathing buffer during stimulation. Four minutes after the loading session, a second round of stimulation resulted in extensive distaining of FM1-43 fluorescence from the cells, which indicated the releasing of the internalized FM1-43. This observation clearly demonstrated the recycling of the endocytosed membrane [34]. Although derived from rat adrenal chromaffin cells, PC12 cells are different from adrenal chromaffin cells in that adrenal chromaffin cells contain mostly dense core vesicles. In PC12 cells, both dense core vesicles and small synaptic like vesicles exist, there are two possible forms of vesicle recycling; and the observed second round of releasing of FM1-43 labeled vesicles (Fig. 3) was contributed by either if not both types of recycling vesicles. Since, compared with control, no difference was observed in the recycling of the endocytic membrane in SIP30 siRNA transfected PC12 cells, SIP30 is not involved in the recycling of either dense core vesicles or small synaptic like vesicles.

The reduction in the releasing rate constant K of exocytosed vesicles reflects the reduced speed in preparing the vesicles into readily releasable status and/or the final releasing, in which multiple steps are involved and multiple molecular players participate. In the current study, the involvement of SIP30 in vesicle exocytosis is clearly demonstrated. As a SNAP25 interacting protein, it is highly likely that SIP30 functions as a regulator of vesicle exocytosis through its interaction with SNAP25, which is suggested by the reduced expression of SNAP25 by SIP30 inhibition [18]. Another study has discovered an interaction between SIP30 and Rab3 proteins [21], which are involved in the regulation of releasing probability of readily releasable vesicles [35]. This indicated another possible cellular mechanism by which SIP30 may be involved in vesicle exocytosis. SIP30 contains a putative coiled-coil domain, which is important for its interaction with both SNAP25 and Rab3 [21]. The coiled-coil domain is the only identifiable motif in SIP30, hence we hypothesize that SIP30 functions as a SNARE protein in the synaptic exocytosis process by docking with its interaction partners such as SNAP25 and Rab3, relaying the vesicle fusion signal to its partners, and influencing the exocytosis process. Such docking capacity likely underscores SIP30 involvement for its ability to modulate neuropathic pain.

## Funding

This work was supported in part by grants from the National Institutes of Health of the United States (DA013471).

## CRediT authorship contribution statement

**Ning Guo:** Writing – review & editing, Writing – original draft, Methodology, Investigation, Formal analysis, Data curation, Conceptualization. **Lei Yu:** Writing – review & editing, Writing – original draft, Supervision, Resources, Project administration, Funding acquisition, Conceptualization.

## Declaration of competing interest

The authors declare no conflict of interest.
